# A Comparative Analysis of Polysaccharides and Ethanolic Extracts from Two Egyptian Sweet Potato Cultivars, Abees and A 195: Chemical Characterization and Immunostimulant Activities

**DOI:** 10.3390/metabo14040222

**Published:** 2024-04-14

**Authors:** Rehab M. Elgabry, Mariam Hassan, Ghada A. Fawzy, Khaled M. Meselhy, Osama G. Mohamed, Areej M. Al-Taweel, Mohamed S. Sedeek

**Affiliations:** 1 Department of Pharmacognosy, Faculty of Pharmacy, Cairo University, Kasr el Aini St., Cairo 11562, Egypt; rehab.elgabry@pharma.cu.edu.eg (R.M.E.); ghada.ah.fawzy@pharma.cu.edu.eg (G.A.F.); khaled.meselhy@pharma.cu.edu.eg (K.M.M.); osama.mohamed@pharma.cu.edu.eg (O.G.M.); 2 Department of Microbiology and Immunology, Faculty of Pharmacy, Cairo University, Cairo 12613, Egypt; mariam.hassan@pharma.cu.edu.eg; 3 Department of Microbiology and Immunology, Faculty of Pharmacy, Galala University, New Galala City 43511, Egypt; 4 Natural Products Discovery Core, Life Sciences Institute, University of Michigan, Ann Arbor, MI 48109, USA; 5 Department of Pharmacognosy, College of Pharmacy, King Saud University, Riyadh 11495, Saudi Arabia; amaltaweel@ksu.edu.sa

**Keywords:** sweet potato, Abees, active constituents, biological activities, immunostimulant properties, phenolic acids, polysaccharides

## Abstract

Sweet potato (*Ipomoea batatas* (L.) Lam.) belongs to family Convolvulaceae. The plant is distributed worldwide and consumed, especially for its edible tubers. Many studies have proved that the plant has variable biological activities such as antidiabetic, anti-cancer, antihypertensive, antimicrobial, and immunostimulant activities. The roots of sweet potatoes are rich in valuable phytochemical constituents that vary according to the flesh color. Our investigation focused on the chemical profiling of two Egyptian sweet potato cultivars, Abees and A 195, using UPLC-QTOF and the analysis of their polysaccharide fractions by GC-MS. Furthermore, we assessed the immunostimulant properties of these extracts in immunosuppressed mice. The study revealed that sweet potato roots contain significant concentrations of phenolic acids, including caffeoylquinic, caffeic, caffeoyl-feruloyl quinic, and p-coumaric acids, as well as certain flavonoids, such as diosmin, diosmetin, and jaceosidin, and coumarins, such as scopoletin and umbelliferone. Moreover, polysaccharides prepared from both studied cultivars were analyzed using GC-MS. Further biological analysis demonstrated that all the tested extracts possessed immunostimulant properties by elevating the level of WBCs, IL-2, TNF, and IFN-γ in the immunosuppressed mice relative to the control group with the highest values in polysaccharide fractions of A195 (the ethanolic extract showed a higher effect on TNF and IFN-γ, while its polysaccharide fraction exhibited a promising effect on IL-2 and WBCs). In conclusion, the roots of the Egyptian sweet potato cultivars Abees and A 195 demonstrated significant immunostimulant activities, which warrants further investigation through clinical studies.

## 1. Introduction

During the last few decades, there has been an increase in immunodeficient diseases due to air pollution, unhealthy diets, and unhealthy lifestyles (high alcohol intake, lack of physical activity, obesity, smoking, etc.). Disturbances in body immunity can promote the development of serious diseases such as cancer and viral and bacterial infections [1,2]. Different studies have proved that the restoration of the function of the immune system is essential for the successful treatment of different illnesses, which has initiated researchers to look for potential, natural sources of immunomodulators [3]. Plants are considered valuable immunomodulatory agents as they contain various active constituents such as polysaccharides, sterols, alkaloids, lectins, flavonoids, and glycoproteins that enhance human health and the immune system [4]. For example, polysaccharides such as acidic arabinogalactan and rhamnogalacturonan were shown to possess immunostimulatory effects in vivo and in vitro [5].

Sweet potato (SP) is one of the most widely consumed edible plants worldwide. The plant belongs to family Convolvulaceae (Morning Glory family), and China is the leading country in its production [6]. SP is a “poor man’s vegetable crop” in Africa (which is known as “women’s work?”). SP yields the most nutrients and biomass per hectare of any crop on the planet. It is highly adapted for survival in tropical regions and to produce roots without the use of irrigation or fertilizers, and it plays a crucial role in famine treatment. Thousands of towns in East Africa, in plains that are highly populated, rely on SP for food security, and the Japanese employed it when typhoons destroyed the rice crops. Additionally, it is the fourth most consumed crop in Brazil, with a high-energy, carbohydrate-rich diet. It also contains enough amounts of vitamins B complex, C, and A [7]. In Egypt, SP is one of the most highly valuable vegetable crops with an economical value. SP cultivation during 2018 took place on an area of c. 28,525.86 Feddan in Egypt, with an average yield of c. 11.767 Tons/Feddan. Additionally, there has been very little research on the medicinal value of Egyptian SP cultivars. Thus, our aim was to explore the biological activity and medicinal value of SP, hoping to highlight the promising potential of SP as a cheap, available functional food in the Egyptian market [8]. The importance of SP in the human diet has traditionally been a supply of carbohydrates. The edible part of SP is its root, which is rich in many biologically active ingredients such as polysaccharides, phenolic acids, flavonoids, fibers, vitamins, and proteins [5]. Different SP varieties have different flesh colors, like white, yellow, orange, and purple. The color variation is due to variation in chemical composition, as purple SP is rich in anthocyanins and possesses immunostimulant activity [9], while the orange variety contains high levels of β-carotene, which is very crucial in the treatment of vitamin A deficiency [10,11]. Many biological activities were reported for SP polysaccharides, such as anti-tumor [12], immunostimulatory [13] and anti-inflammatory effects [14]. Additionally, SP root fibers are proven to be efficient prebiotic sources [15]. A preclinical study showed that sweet potato fiber extract facilitated IgM production by HB4C5 cells and revealed that it has positive effects on immunostimulatory activity in vitro [16]. SP pulp contains 49.7% of dietary fiber, which is highly rich in pectins (39.5%), lignin, hemi cellulose, and cellulose [17]. A study on purple SP found that anthocyanins and phenolic compounds demonstrated antioxidative activities that might contribute to better B cell functions, macrophages, and T helper cells by inhibiting oxidative stress [18]. Other biological activities of sweet potatoes include anti-cancer, anti-allergy, and improving vaccine efficacy [14,19,20]. Phenolic acids like caffeic, chlorogenic, isochlorogenic, hydroxycinammic, and cinammic acids are present in SP. Phenolic acids are associated with sensory properties, color, nutritional importance, and antioxidant activities of foods [21]; they are more prominent in purple SP than other varieties [22]. Our study aims to explore the chemical composition of two Egyptian SP cultivars, namely Abees and A 195, using UPLC-QTOF and GC-MS and to investigate the immunostimulant activity of their ethanolic extracts and polysaccharide fractions on immunosuppressed mice. It is worth noting that there have been no previous reports on the chemical composition or the immunostimulant activity of these two Egyptian SP cultivars.

## 2. Materials and Methods

### 2.1. Phytochemical Study

#### 2.1.1. Collection of Plant Material

The roots of two Egyptian SP cultivars, namely, “Abees” and “A 195”, were obtained from “The Potato and Vegetables Department, Horticulture Research Institute, Agricultural Research Center, Egypt”. Ten kg of each root were collected, washed with water, and cut into small pieces for drying in the oven at 45 °C for 10 days. The materials were pulverized into fine powder.

#### 2.1.2. Preparation of Extracts and Polysaccharide Fractions

Two kilograms of dried powdered Abees and A 195 cultivars were each extracted with 85% ethanol (6 L) at room temperature for 10 days with frequent shaking to prepare the crude extract, and then the filtrate was concentrated with a rotary evaporator (Buchi, Germany), and the final weight was 100 g of each extract. The residual powders were then redried in the vacuum oven (Thermo Fisher Scientific Inc. Waltham, Massachusetts, U.S) and extracted with hot water at 60 °C for 3 h. The residue was then removed by decantation, and the supernatant was precipitated using absolute ethanol (1:8, *v*/*v*). The crudely precipitated polysaccharides were collected by centrifugation and recrystallized using hot water and ethanol, and then they were kept in the desiccator for analysis at room temperature. All the steps of preparation were applied according to the method of G. Zhao et al. [23], with slight modifications.

#### 2.1.3. UHPLC-QTOF-MS/MS Profiling of Ethanolic Extracts

Ultra-high-performance liquid chromatograms (UHPLCs) obtained by an Agilent LC–MS system composed of an Agilent 1290 Infinity II UHPLC are coupled to an Agilent 6545 ESI-Q-TOF-MS in both positive and negative modes. Aliquots (1 µL) of ethanolic extracts (2 mg/mL in MeOH),were analyzed on a Kinetex phenyl-hexyl (1.7 µm, 2.1 × 50 mm) column and then eluted with isocratic elution of 90% A (A: 0.1% formic acid +100% H_2_O) for 1 min, followed by gradient elution to 100% B (5% H_2_O +95% MeCN + 0.1% formic acid) for 6 min with a flow rate of 0.4 mL/min. The ESI conditions were established at a capillary temperature of 320 °C, a sheath gas flow rate of 11 L/min, and a source voltage of 3.5 kV. Ions were detected in the full scan at 6 scans/s with an intensity above 1000 counts, an isolation width of 1.3~*m*/*z*, a maximum of 9 selected precursors per cycle, and using ramped collision energy (5 × *m*/*z*/100 + 10 eV). Purine C_5_H_4_N_4_ [M + H] + ion (*m*/*z* 121.050873) and hexakis (1H,1H,3H-tetrafluoropropoxy)-phosphazene C_18_H_18_F_24_N_3_O_6_P_3_ [M + H] + ion (*m*/*z* 922.009798) were used as internal lock masses for the positive mode, while TFA C_2_HF_3_O_2_[M − H] − ion (*m*/*z* 112.985587) and hexakis (1H,1H,3H-tetrafluoropropoxy)-phosphazene C_18_H_18_F_24_N_3_O_6_P_3_ [M + TFA − H] − ion (*m*/*z* 1033.988109) were used as internal lock masses for the negative mode. Only peak list rows filter features with an accompanying MS2 data and their retention time, which is between 0 and 9.0 min were kept. (vii) Duplicate peak filter: filter mode, old average; *m*/*z* tolerance, 0.02 *m*/*z*; RT tolerance, 0.5 min. [24]. Data acquisition (2.5 Hz) in the profile mode was governed by MassHunter Workstation software (version B.04.00, Agilent technologies, Santa Clara, CA, USA). The spectra were acquired in positive and negative ionization modes over a mass-to-charge (*m*/*z*) range from 70 to 1100. The tentative identification was performed by selecting the major peaks in the chromatograms, choosing the molecular formula, which has a higher probability and lower error ratio, and comparing the fragmentation spectrum of each identified compound in previous studies. The detection window was set to be 100 ppm. The tentative identification of compounds was performed by the generation of the candidate formula with a mass accuracy limit of 10 ppm, considering RT, MS2 data, and the reference literature.

#### 2.1.4. Gas Chromatography Mass Spectroscopy (GC-MS) Analysis of the Polysaccharides

The monosaccharide composition of the polysaccharides of the two cultivars was analyzed using GC-MS. A total of 500 mg of crude polysaccharide from each cultivar was dissolved in 1 mL of TFA (2 mol/L) and then hydrolyzed by microwave-assisted hydrolysis at 100 °C. The microwave irradiation program was set for 6 min at 500 W. The solution was dried under a stream of nitrogen gas. Subsequently, the hydrolysates were prepared for the derivatization procedure. A total of 0.5 mL of each hydrolysate was evaporated to dryness in small screw-topped septum vials at 40 °C under a stream of nitrogen. When they were almost dry, 0.5 mL of isopropanol was added, and drying under a stream of nitrogen was completed until a dry, solid residue was obtained. We then screwed on the septum and injected 0.5 mL of oxamination reagent (2.5% hydroxylamine hydrochloride in pyridine). We mixed and heated it at 80 °C for 30 min and then allowed it to cool. We injected 1 mL of silylating reagent (N, O-bis-(trimethylsilyl) acetamide: trimethylchlorosilane, 5:1 by volume. We mixed and heated it at 80 °C for 30 min then allowed it to cool [25]. Analysis was performed using the Shimadzu GCMS-QP2010 (Tokyo, Japan) equipped with an Rtx-5MS fused bonded column (30 m × 0.25 mm i.d. × 0.25 µm film thickness) (Restek, Bellefonte, PA, USA) equipped with a split–splitless injector. The initial column temperature was kept for 2 min at 45 °C, then programmed to 300 °C at the rate of 5 °C/min and kept constant for 5 min at 300 °C . The injector temperature was set to 250 °C. The flow rate of helium carrier gas was 1.41 mL/min. All mass spectra were recorded by applying the following conditions: (equipment current) filament emission current, 60 mA; ionization voltage, 70 eV; ion source, 200 °C. Diluted samples (1% *v*/*v*) were injected in split mode (split ratio 1:15).

### 2.2. Immunostimulant Activity of the Ethanolic Extracts and Polysaccharide Fractions

#### 2.2.1. Ethics Statement

All animal procedures were approved by the Research Ethics Committee of the Faculty of Pharmacy, Cairo University (Approval no. MP (3308)) following the Guide for the Care and Use of Laboratory Animals published by the Institute of Laboratory Animal Research (USA).

#### 2.2.2. In Vivo Evaluation of the Immunostimulant Activity of the Ethanolic Extracts and Polysaccharide Fractions

The immunostimulant activity of the tested extracts was investigated as described before [26,27]. Male Balb/c mice were purchased at 4 weeks old with an average weight of 22 ± 3.7 gm. Mice were housed six per cage and were allowed to be acclimated for 1 week (at 25 ± 2 °C, with the 12:12 h dark/light regime) before starting the experiment. Animals were supplied with standard commercial food and tap water ad libitum. Mice were divided randomly into six groups (six mice per group, n = 6) (Table 1). All the treatments of extracts were administered orally (p.o.) using an oral gavage once daily for five weeks (Table 1), during which the mice body weights were measured once a week. All the groups except the normal control (healthy control) group were treated intra-peritoneally (i.p.) with cyclophosphamide (CYP) at a dose of 70 mg kg 1 for 3 days during the last week of the experiment [27]. Blood samples were taken from the animals of the experimental groups on the last day of the experiment by retro-orbital puncture. Hematological parameters were studied for hemoglobin levels, RBC, platelets, and total WBC count [28]. For sera collection, the blood was centrifuged at 4000 rpm for 10 min. The upper serum of each tube was collected and stored at −80 °C till biomarker analysis. Enzyme-linked immunosorbent assay (ELISA) was performed for the quantification of levels of IL-2, TNF-α, and IFN-γ in the mice sera using the “LifeSpan Biosciences™ Mouse Interleukin IL-2 ELISA Kit—LS-F5102”, “LifeSpan Biosciences™ Mouse Interleukin TNF-α ELISA Kit—LS-F2559”, and “LifeSpan Biosciences™ Mouse IFN (gamma) ELISA Kit—LS-F3414” (LifeSpan Biosciences, Shirley, MA, USA), respectively, according to the manufacturer’s instructions [29].

## 3. Results

### 3.1. Phytochemical Study

#### 3.1.1. UPLC-MS/MS Analysis

**Figure 1:**
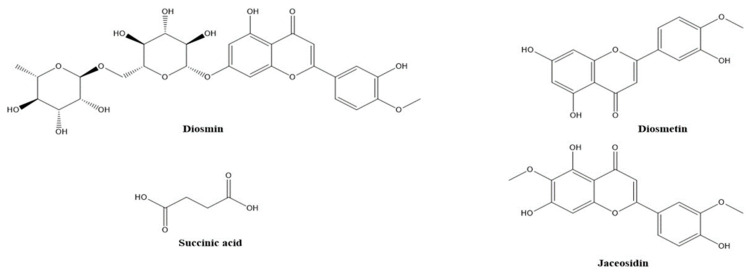
Chemical structures of some phenolic metabolites only detected in A 195 cultivar.

**Figure 2:**
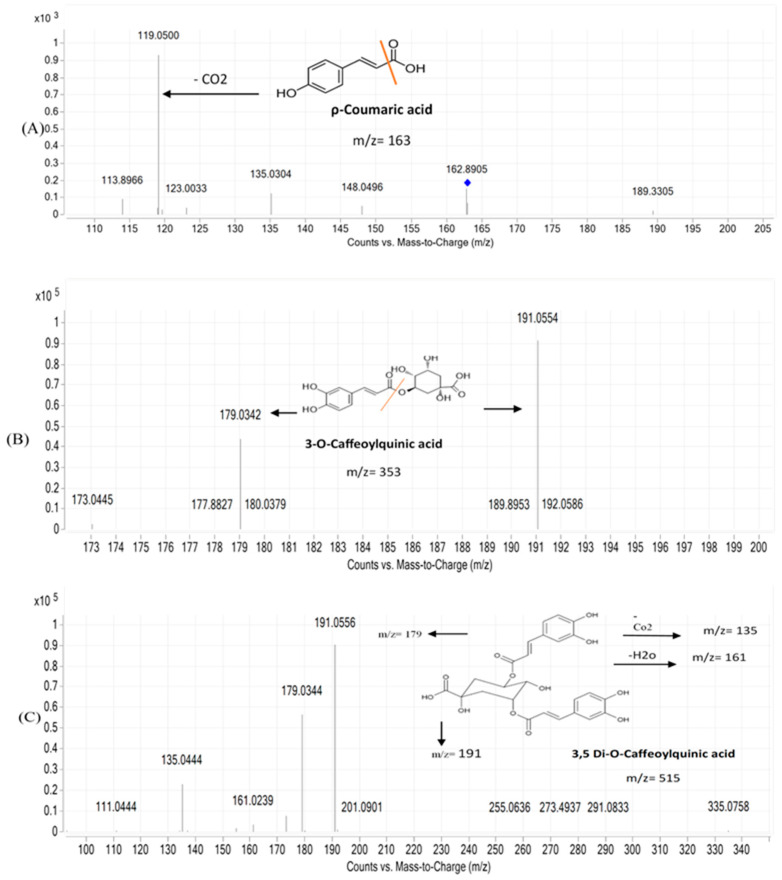
MS/MS fragmentation pattern of selected metabolites in Abees and A 195 cultivars. (**A**), p-coumaric acid: (**B**), 3-*O*-caffeoylquinic acid; (**C**), 3,5 dicaffeoylquinic acid, blue dot indicates the molecular weight suggested by the software, orange line was drawn to indicate site of fragmentation.

The presence of numerous compounds or metabolites was detected by analyzing UPLC-MS chromatograms. Several isomers were recognized by comparing their retention times and fragmentation patterns to those of comparable substances previously examined under similar conditions. Many phenolic chemicals were discovered using this UPLC-MS technique. Thirty constituents, including phenolic acids, carboxylic acids, coumarins, and flavonoids were tentatively identified in the ethanolic extracts using mass spectral data and retention time. The molecular formula, retention time, and mass fragments of the tentatively identified compounds are shown in Table 2. The disaccharide sucrose compound (peak 1) was identified by the precursor ion peak at *m*/*z* 341.1100 and its fragments 179. 0314, 161.0233, and 101.0233 with a molecular formula of C_12_H_22_O_11_. Quinic acid and feruloyl quinic acid peaks were detected by their precursor ion peaks at *m*/*z* 191.0550 and 367.1020, respectively. The caffeic acid peak was tentatively identified by its precursor ion at *m*/*z* 179.0300 [M-H] and base peak at *m*/*z* 135.0454 after the loss of the COO group 135 [M-H]. Peaks 5 and 7, which represent protocatechuic acid and protocatechualdehyde, were detected at retention time (0.850, 1.216 min) by their precursor ions at *m*/*z* 153.0100 and 137.0200 [M-H], respectively. Protocatechuic acid major fragment appears at [M-H] 109.0286 due to loss of the COO group; the base peak of protocatechualdehyde was detected at retention time 1.2 min with a fragment ion 119.0117 after loss of the CO group. Two coumarin derivatives, scopoletin (peak 8) and umbelliferon (peak 16), were tentatively identified by their precursor ion peaks at *m*/*z* 193.0410 and 163.0300 [M+H], respectively, in a positive mode. The two major fragments of scopoletin 178.0237 and 133.0577 are caused by the loss of CH_3_ and COCH_3_OH, respectively. Peak 21 has similar parent ion peaks at *m*/*z* 353.0850 [M-H]. It was assigned as mono-CQAs with the formula C_16_H_18_O_9_ and tentatively identified as 3-O-caffeoylquinic acid with a precursor ion peak [M-H] at *m*/*z* 353.0868. After fragmentation, there are two major fragments: 191.0559 for quinic acid and 179.0350 for caffeic acid. Five types of di-CQAs with a deprotonated [M-H] at *m*/*z* 515.1100 and a typical fragment ion at *m*/*z* 353.0830 were identified to be 3,4-di-O-caffeoylquinic acid (peak 17), 3,5-di-O-caffeoylquinic acid (peak 18), 1,3 Di caffeoylquinic acid (peak 19), 1,5-Dicaffeoylquinic acid (peak 20), and 4,5-di-O-caffeoylquinic acid (peak 22), respectively. Generally, dicaffeoylquinic acids have a similar fragmentation pattern by losing one caffeic acid with *m*/*z* at 179 and monocaffeolquinic acid at *m*/*z* 353, then fragmentation of monocaffeolquinic acid into caffeic acid and quinic acid with *m*/*z* 179 and 191, respectively. P-coumaric acid peak was tentatively identified at precursor ion peaks at *m*/*z* 163.0310 [M-H] with a fragment ion *m*/*z* 135.0304 [M-H] and has a molecular formula of C_9_H_8_O_3_. Figure 1 showed the chemical structure of Some phenolic compounds were found in A 195 cultivar, including succinic acid and the other three flavonoids (diosmin, diosmetin, and jaceosidin). Succinic acid is a dicarboxylic acid; its precursor ion peak was detected at *m*/*z* 117.0178 due to loss of the H_2_O molecule, resulting in a fragment 99.9249, and loss of the COO group gives a fragment 73.0289. Diosmin, which is a flavone glycoside, and its aglycone diosmetin were identified with parent ion peaks at *m*/*z* 607.1660 and 299.0565; after breakage, the glycosidic bond of diosmin gives a major fragment of diosmetin at *m*/*z* 299.0565 and a retention time of 4.015. Jaceosidin is a trihydroxyflavone aglycone, and its precursor ion peak was detected at *m*/*z* 329.0671 [M-H] after fragmentation and a loss of the OCH_3_ group, resulting in a major peak of diosmetin at *m*/*z* 299.0196 and a loss of the CH_3_ group, which gave a fragment peak at *m*/*z* 314.0432. Figure 2 showed the fragmentation pattern of some metabolites found in Abees and A 195 cultivars. Table S1 showed the MS/MS fragmentation pattern of some tentatively identified compounds using Mass Hunter software program.

#### 3.1.2. Chemical Characterization of Polysaccharides Fractions

polysaccharides hydrolysate was obtained from two cultivars, namely “Abees” and “A 195”, which were analyzed using GC-MS. The presence of monosaccharides was verified using NIST library by comparing the retention index and fragmentation pattern [37]. The resulting compounds and their corresponding area percentages are presented in Table 3.

As summarized in Table 3, the polysaccharides hydrolysate of the two cultivars are mainly composed of glucose, arabinose, rhamnose, gluconic acid, psicose, galactose, galacturonic acid, and glucaric acid with different ratios. Some differences exist in the monosaccharide composition between Abees and A 195 cultivars. For example, Abees contains some monosaccharides that are not found in A 195, such as D-fructofuranose, D-galactopyranose, fructose oxime, and D-xylopyranose. The concentration of glucose is significantly higher in “A 195” (13.88) than in “Abees” (4.04). The difference in polysaccharide composition may contribute to the higher immunostimulant effect of A 195, this agrees with a previous study that was conducted on a polysaccharide isolated from *Gracilaria lemaneiformis* to investigate its immunomodulatory activity. The results showed that the polysaccharide with a higher concentration of glucose improved the pinocytic capability and proliferation of RAW264.7 cells and promoted the production of tumor necrosis factor-α (TNF-α) and interleukin-6 (IL-6) by activating iNOS, IL-6, and TNF-α gene expressions [38]. Another immunomodulatory study performed on the polysaccharide isolated from *Angelica sinensis* demonstrated its immune-enhancing effects, and it was composed of arabinose, rhamnose, glucose, mannose, and galactose with a higher level of glucose [31]. Moreover, L-gluconic acid, D-glucuronic acid, and galacturonic acid were only detected in A 195. According to some previous studies, these compounds could potentiate the immune-enhancing properties of A 195 polysaccharide [39,40].

### 3.2. Imunostimulant Activity of the Ethanolic Extracts and Polysaccharides Fractions

**Figure 3:**
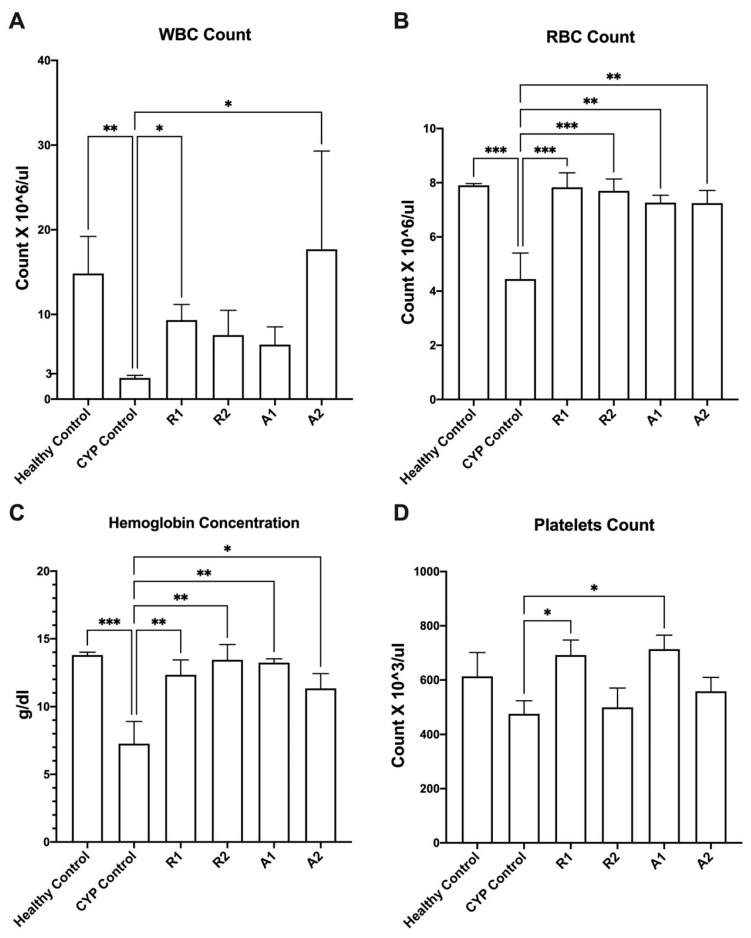
Effect of the tested botanical extracts on white blood cell (WBC) count (**A**), red blood cell (RBC) count (**B**), hemoglobin concentration, (**C**) and platelets count (**D**) in immunosuppressed mice. Healthy control group was treated with the vehicle (distilled water). CYP control group was treated with cyclophosphamide (70 mg kg^−1^, i.p.). R1, R2, A1, and A2 groups were treated with ethanolic extract of Abees (450 mg kg^−1^, orally), ethanolic extract of A 195 (450 mg kg^−1^, orally), polysaccharide fraction of Abees (450 mg kg^−1^, orally), and polysaccharide fraction of A 195 (450 mg kg^−1^, orally), respectively. *, ** and *** indicate that the difference is significant at *p* < 0.05, 0.01, and 0.001, respectively (one-way ANOVA, Fisher’s multiple comparison test).

A mice model for studying the immunostimulant activity of the tested extracts has been successfully developed and validated. Male Balb/c mice were randomly separated into six groups (six mice per group). Four groups of mice were daily administered the tested extracts/fractions (450 mg kg^−1^ p.o.) for five weeks. Two control groups were employed; the normal control (healthy control) group was administered only the vehicle (distilled water), and the immunosuppressed control (CYP control) group was administered the vehicle and treated with cyclophosphamide. The administration of cyclophosphamide (CYP) (70 mg kg^−1^ for three days i.p.) succeeded in achieving immunosuppression in the CYP control group, represented by the significant inhibition in red blood cell count, white blood cell count, and hemoglobin concentration when compared to the healthy control group (Figure 3) (one-way ANOVA, Fisher’s multiple comparison test, *p* < 0.05). Interestingly, the tested extracts/fractions alleviated the cyclophosphamide-induced immunosuppression in mice, as indicated by increased levels of WBC, RBC, hemoglobin, and platelets when compared to the CYP control group (Figure 3). Abees ethanolic extract and A 195 polysaccharide fraction recorded significantly higher levels of WBC counts than the control group in the immunosuppressed mice (Figure 3A) (one-way ANOVA, Fisher’s multiple comparison test, *p* < 0.05). All the tested extracts recorded significantly higher levels of RBC counts and hemoglobin concentrations than the control group in the immunosuppressed mice (Figure 3B, C) (one-way ANOVA, Fisher’s multiple comparison test, *p* < 0.05). Both the ethanolic extract and polysaccharide fraction of the Abees cultivar recorded significantly higher levels of platelet counts than the control group in the immunosuppressed mice (Figure 3D) (one-way ANOVA, Fisher’s multiple comparison test, *p* < 0.05).

**Figure 4:**
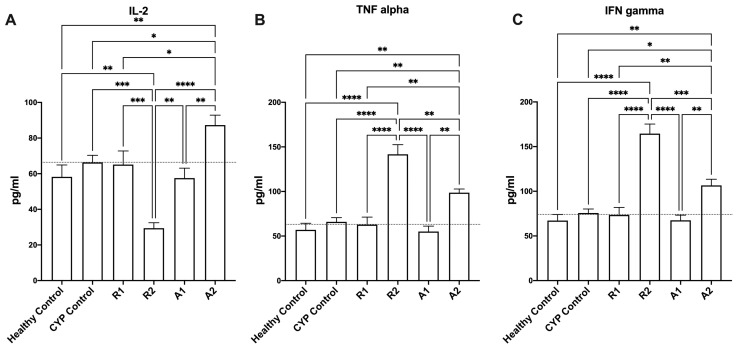
Effect of the tested botanical extracts on IL-2 (**A**), TNF-α (**B**), and IFN-γ (**C**) in immunosuppressed mice. Healthy control group was treated with the vehicle (distilled water). CYP control group was treated with cyclophosphamide (70 mg kg^−1^, i.p.). R1, R2, A1, and A2 groups were treated with ethanolic extract of Abees (450 mg kg^−1^, orally), ethanolic extract of A 195 (450 mg kg^−1^, orally), polysaccharide fraction of Abees (450 mg kg^−1^, orally), and polysaccharide fraction of A 195 (450 mg kg^−1^, orally), respectively. *, **, ***, and **** showed that the difference is significant at *p* < 0.05, 0.01, 0.001, and 0.0001, respectively (one-way ANOVA, Fisher’s multiple comparison test).

Oral administration of the tested extracts modulated the levels of TNF-α, IL-2, and IFN-γ in the immunosuppressed groups (Figure 4). Interestingly, the administration of A 195 polysaccharide fraction showed the best immunostimulant activity, with a significant promotion in the production of different immunity-related mediators such as IL-2, TNF-α, and IFN-γ in the immunosuppressed mice when compared to the CYP control group (Figure 4A–C) (one-way ANOVA, Fisher’s multiple comparison test, *p* < 0.05). It is also worth mentioning that the administration of the A 195 ethanolic extract was accompanied by significantly higher levels of TNF-α and IFN-γ in the immunosuppressed mice when compared to the CYP control group (Figure 4B,C) (one-way ANOVA, Fisher’s multiple comparison test, *p* < 0.05).

## 4. Discussion

Sweet potato comes in seventh among almost all food crops globally, with about 115 million metric tons of production annually. Sweet potato is rich in nutrients, providing about 90% of the nutrients required by most people worldwide [41]. The roots of sweet potatoes are rich in phenolic compounds, polysaccharides, fibers, and vitamins [42]. Previous studies have proved that polyphenols, such as phenolic acids and flavonoids, affect human health and enhance the body’s immune system [43]. Oral administration of green tea as an example of a polyphenol-rich medicinal plant improved phagocytosis, prevented depletion in the number of antigen-presenting cells, and prevented ultraviolet-induced infiltration of leukocytes in mice [44]. Additionally, polysaccharides were found to enhance the immune response. For example, polysaccharides, which come from sugar cane, demonstrated immunostimulant properties and activated the classical complement pathway in the body’s serum by interacting with immunoglobulins [43]. Our comparative study investigated the chemical profiling of Egyptian Abees and A 195 sweet potato cultivars (two ethanolic extracts and two polysaccharide fractions) and their immunostimulant effects on the immunosuppressed male Balb/c mouse model. UPLC-HRESIMS results of the two cultivars showed that the ethanolic extracts of Abees and A 195 roots mainly composed of phenolic acids (caffeoylquinic acids, caffeic acid, protocatechuic acid, ρ-coumaric acid, caffeoyl feruloyl quinic acid, and others), coumarins (umbelliferone and scopoletin), and flavonoids (diosmin, diosmetin, and jaceosidin). Previous studies proved that phenolic acids have immunostimulant activities; for example, caffeic and p-coumaric acids significantly enhance the proliferation of LPS-stimulated splenocytes, suggesting the potential activation of B cells and the promotion of the humoral immune system in hosts [45]. In a study conducted on *Gallium aparine* (L.), it was reported that increasing the amount of phenolics (flavonoids and hydroxycinnamic acid derivatives) in the tested extracts and lowering the iridoids content in 60% and 96% EtOH when compared to 20% EtOH suggested that polyphenolic compounds can be responsible for the reported immunostimulant activities [46]. Our investigation revealed that both cultivars contain significant amounts of caffeoylquinic acids, which are prominent phenolic components and may have a crucial role in the immune-enhancing effects of Abees and A 195 cultivars. Based on our findings, it can be suggested that phenolic compounds in both ethanolic extracts of Abees and A 195 are responsible for the observed immunostimulant properties as they potentiate the immunological parameters in immunosuppressed mice. In a previous GC-MS analysis, the polysaccharide chain of sweet potatoes was identified as (1-6)-α-D-glucan. Other studies have demonstrated the immunostimulant activity of (1-4)-α-glucan [47]. Moreover, (1-6)-α-D-glucan polysaccharides isolated from sweet potatoes were found to have immunostimulatory activities by increasing macrophage phagocytic functions, splenic lymphocyte proliferation, and IgG concentration in serum [23]. A study conducted on *Echinacea purpurea* reported that its polysaccharides have a potential enhancing property on the immune system [48]. It was reported that the immunostimulatory properties of plant extracts are usually attributed to the presence of polysaccharides [49]. An experiment conducted to investigate the anti-cancer and immunomodulatory activities of polysaccharides found that polysaccharides potentiated the macrophages release of cell factors, TNF-a, NO, and PGE2. Furthermore, numerous studies have demonstrated that specific polysaccharides can enhance body immune functions, such as macrophages, monocytes, granulocytes, and NK cells, and stimulate the secretion of IFN-c, IL-6, and IL-8 [50]. These findings support our results on the immunological effects of Abees and A 195 polysaccharides, as both fractions were reported to stimulate immunological parameters in immunosuppressed mice compared to the control group.

In this study, the effects of two ethanolic extracts and two polysaccharide fractions were investigated on immunosuppressed mice using a complete blood cell count to detect their impact on WBCs, RBCs, hemoglobin, and platelets, using an ELISA analysis to measure the effect on the immunological parameters IL-2, TNF-α, and IFN-γ. Our results showed that all the extracts and fractions modulate cyclophosphamide-induced immunosuppression in mice, as evidenced by increased or normalized levels of WBCs, RBCs, hemoglobin, platelets, IL-2, TNF, and IFN-γ when compared to the CYP control group and healthy control group. To explain the results of the ELISA analysis on IL-2, TNF-α, and IFN-γ parameters, it is vital to understand their roles in immune responses. The release of antibodies against T cell-dependent antigens needs the contribution of macrophages and dendritic cells as antigen-presenting cells (APCs), the identification of antigenic peptides by helper T cells (Th), and the production of cytokines like interferon (IFN)-g and interleukin (IL)-2 that contribute to the activation of B cells [19]. Immunomodulatory plant extracts can indirectly stimulate hematopoiesis via modulation of the immune response, but the mechanism of this process can be complex and multifactorial. The immune system plays a crucial role in regulating hematopoiesis, and the immunomodulatory extract can influence the production and activity of growth factors and cytokines involved in hematopoiesis and hence stimulate the process; this was observed with sweet potato extract in our study and Ginseng extract in a previous study [51]. Therefore, increasing the levels of these cytokines is crucial for a robust immune response, and our results align with this notion, as the administration of tested extracts and fractions orally modulated the levels of TNF-α, IL-2 and IFN-γ in the immunosuppressed mice. Interestingly, the administration of A 195 polysaccharide fraction and ethanolic extract showed higher immunostimulant activity. As shown in (Figure 4B) there is a higher promotion in the production of TNF-α in the A 195 polysaccharide fraction group (98.7 ± 4.1 pg/mL) and the A 195 ethanolic extract group (141.7 ± 10.7 pg/mL) compared to the healthy (56.9 ± 7.4 pg/mL) and the CYP control (65.9 ± 4.8 pg/mL) groups. Additionally, in (Figure 4C) the mean values of IFN-γ in the immunosuppressed mice treated with the A 195 ethanolic extract (164.5 ± 10.7 pg/mL) and the A 195 polysaccharide fraction (107.6 ± 6.9 pg/mL) were the highest compared to the CYP control group (75.6 ± 4.5 pg/mL). Administration of the A 195 polysaccharide fraction gave the highest promotion in IL-2 values (87.23 ± 5.6 pg/mL). The superior immunostimulant effect of A 195 extracts may be attributed to differences in the chemical composition of their ethanolic and polysaccharide fractions compared to the Abees cultivar. Specifically, the ethanolic extract of A 195 contains certain phenolic compounds, such as diosmin, diosmetin, jaceosidin, and succinic acid, which are absent in the Abees cultivar. The chemical compounds presented in Figure 1 are only detected in the A 195 cultivar. Diosmin flavonoid and its flavone aglycone diosmetin are among the phenolic compounds with valuable biological activities. A study conducted on healthy nonsmokers investigating the biological activity of some flavonoids, including diosmin and diosmetin, and others showed that they exhibited anti-inflammatory and immunomodulatory activities [52]. Furthermore, numerous in vivo and in vitro studies have validated the extensive range of diosmin’s biological properties, which present antihyperglycemic, antioxidative, antimutagenic, anti-inflammatory, and anti-ulcer activities. Jaceosidin, a bioactive flavone, is a potentially active metabolite that possesses a variety of pharmacological effects, including antioxidant, anti-inflammatory, anti-bacterial, anti-cancer, and antiallergic activities [52,53] The bioactivities and therapeutic effects of jaceosidin also include the modulation of different cell signaling pathways (ERK1/2, NF-κB, PI3K/Akt, and ATM-Chk1/2) that have been disturbed in various pathological diseases. A previous study applied to *Artemisia argyi* ethanolic extract, which is rich in some unique flavones such as eupatilin and jaceosidin, displayed a significant immunomodulatory effect [54]. Succinic acid also has immune enhancing properties [55]. Various preclinical studies have consistently demonstrated the immunostimulant effects of sweet potato, with the activity attributed to active constituents such as anthocyanins, phenolic compounds, polysaccharides, and fibers [9,12,16]. Our study on Egyptian sweet potato cultivars aligns with some of these studies by measuring the same cytokine levels (IL-2, TNF-α, and IFN-γ) and confirming the immunostimulant activity through modulation of these specific cytokines [29]. Additionally, other studies have reported immunostimulant effects of purple sweet potato using different immunological parameters, including enhancements in splenocytes and thymocytes proliferation, prevention of T- and B-cell proliferation suppression, elevated serum IgG concentrations, and enhanced lymphocyte proliferation [11,23]. Our findings are consistent with prior research, revealing the immunostimulant properties of the ethanolic extract and polysaccharide fractions of two Egyptian sweet potato cultivars, Abees and A 195. The tentative identification of phenolic acids, flavonoids, and coumarins in the UPLC-MS analysis of their extracts suggested that they are the primary contributors to the immunostimulant effect. Furthermore, the potent effect of the polysaccharide content confirmed that sweet potato functional foods’ immunostimulant effect is enhanced by their polysaccharide content.

## 5. Conclusions

In our study, the ethanolic extracts of two Egyptian sweet potato cultivars, Abees and A 195, were chemically profiled, and it was found that they were mainly composed of phenolic compounds such as phenolic acids, with a high percentage of caffeoylquinic acids. Polysaccharides were extracted from the two cultivars and analyzed by GC-MS. Furthermore, we investigated the immunostimulant activity of both the ethanolic and polysaccharide extracts on immunosuppressed mice. These extracts exhibited immunostimulant activity by increasing the levels of WBCs and RBCs in blood and TNF, IL-2, and IFN-γ in serum samples. Notably, the ethanolic and polysaccharide extracts of the A 195 cultivar showed a higher increase in IL-2, TNF, and IFN-γ levels, indicating a potentially stronger immunostimulant activity than the Abees cultivar.

Finally, the immunostimulant activity of sweet potato extracts has various potential applications in the field of medicine. For example, it can be used as a natural alternative or supplement to conventional immunostimulant treatments, which often have adverse effects and may be costly. Sweet potato extracts could also help address the growing problem of antibiotic resistance, as the immunostimulant can help enhance the body’s natural defences against infections. The ability of sweet potato extracts to stimulate the release of cytokines and enhance the function of immune cells could also be helpful in improving the efficacy of vaccines, as vaccines rely on the immune system to mount a response against the targeted pathogen. Overall, the immunostimulant activity of sweet potato extracts has various potential applications in medicine, and further research is needed to explore their full therapeutic potential.

## Figures and Tables

**Table 1:** Group classification of the mice.

Group (n = 6)	Treatment
A (healthy control)	Vehicle treatment (distilled water)
B (Model control)	Cyclophosphamide (70 mg kg^−1^/i.p.)
C (ethanolic extract of Abees)	R1 extract (450 mg kg^−1^/orally)
D (ethanolic extract of A 195)	R2 extract (450 mg kg^−1^/orally)
E (polysaccharide fraction of Abees)	A1 extract (450 mg kg^−1^/orally)
F (polysaccharide fraction of A195)	A2 extract (450 mg kg^−1^/orally)

**Table 2:** Chemical compounds tentatively identified in the ethanolic extracts.

No.	Compound	Rt (min)	Molecular Formula	(M+H)/(M-H)	Fragments	Error	Abees	A 195	Ref.
1	Sucrose	0.434	C_12_H_22_O_11_	341.1100	179.0314	−1.3	+	+	[30]
					161.0233				
					101.0233				
					59.0141				
2	Quinic acid	0.489	C_7_H_12_O_6_	191.0553	191.0553	−1.91	+	+	[30]
					85.0291				
					59.0141				
3	Succinic acid	0.517	C_4_H_6_O_4_	117.0178	73.0289	−2.52	−	+	[30]
					99.9249				
4	Caffeic acid	0.831	C_9_H_8_O_4_	179.0341/181.0492	135.0454	1.28	+	+	[30]
5	Protocatechuic acid	0.850	C_7_H_6_O_4_	153.0189	109.0286	3.12	+	+	[31]
6	Salicylic acid	1.189	C_7_H_6_O_3_	137.0241	93.3250	1.97	+	+	[30]
7	Protocatechualdehyde	1.216	C_7_H_6_O_3_	137.0240	119.0117	3.07	+	+	[31]
					108.0196				
					93.0339				
8	Scopoletin	1.442	C_10_H_8_O_4_	193.0498	178.0237	−1.27	+	+	[32]
					150.0293				
					133.0577				
9	4-Acetylphenylcaffeic acid	1.577	C_14_H_18_O_7_	297.0980	179.0381	−3.31	+	+	[31]
					161.0250				
					135.0452				
10	3-O-ρ-coumaroyl quinic acid	2.214	C_16_H_18_O_8_	337.0915/339.1067	191.0588	2.87	+	+	[31]
					163.0385				
11	Feruloyl quinic acid	2.408	C_17_H_20_O_9_	367.1021	193.0490	3.82	+	+	[30]
					191.0527				
					173.0447				
12	ρ-Coumaric acid	2.410	C_9_H_8_O_3_	163.0392	119.0500	5.59	+	+	[31]
					135.0304				
13	Diosmin	2.519	C_28_H_32_O_15_	607.1660	299.1540	0.72	−	+	[33]
14	4-Hydroxybenzoic acid	2.740	C_7_H_6_O_3_	137.0238	93.0336	4.6	+	+	[34]
					65.0381				
15	Azelaic acid	2.879	C_9_H_16_O_4_	187.0967	125.0969	−2.74	+	+	[30]
					97.0642				
16	Umbelliferone	2.938	C_9_H_6_O_3_	163.0388	135.0434	0.58	+	+	[32]
					117.0327				
					89.0386				
					107.0480				
17	3,4-di-O-caffeoylquinic acid	2.999	C_25_H_24_O_12_	515.1185/517.1346	353.0830	2.53	+	+	[30]
					335.0735				
18	3,5-di-O-caffeoylquinic acid	3.046	C_25_H_24_O_12_	515.1179	353.0870	2.03	+	+	[30]
					191.0553				
					179.0341				
19	1,3-Dicaffeoylquinic acid	3.073	C_25_H_24_O_12_	515.1188	335.0754	1.6	+	+	[35]
					179.0342				
20	1,5-Dicaffeoylquinic acid	3.088	C_25_H_24_O_12_	515.1187	335.5230	2.05	+	+	[35]
					179.5046				
21	3-O-Caffeoylquinic acid	3.100	C_16_H_18_O_9_	353.0868	191.0559	2.13	+	+	[35]
					179.0350				
					173.0455				
					135.0452				
22	4,5-di-O-caffeoylquinic acid	3.212	C_25_H_24_O_12_	515.1179	353.0854	3	+	+	[30]
					299.1596				
					179.0341				
23	N-trans-p-coumaroyltyramine	3.406	C_17_H_17_NO_3_	282.1124	162.0553	3.19	+	+	[30]
					145.0285				
					119.0494				
24	Caffeoyl-feruloyl quinic acid	3.433	C_26_H_26_O_12_	529.1341	367.1016	2.11	+	+	[30]
					193.0495				
					179.0342				
					173.0446				
25	3,4Dimethoxycinnamic acid	3.571	C_11_H_12_O_4_	207.0655/209.0809	207.0653	3.61	+	+	[31]
					163.7717				
					135.0446				
					133.0289				
26	Trihydroxy-10,15-octadecadienoic acid	3.599	C_18_H_32_O_5_	327.2163	291.1937	2.8	+	+	[30]
					229.1438				
					211.1325				
					171.1014				
27	Diosmetin	4.015	C_16_H_12_O_6_	299.0565	255.0296	−1.19	−	+	[33]
					284.0333				
28	Jaceosidin	4.042	C_17_H_14_O_7_	329.0671/331.0814	299.0196	−0.68	−	+	[33]
					314.0432				
29	Trihydroxy-10-octadecenoic acid	4.043	C_18_H_34_O_5_	329.2334	329.2326	−0.18	+	+	[30]
					311.2222				
					293.2110				
					229.1442				
					211.1336				
					171.1024				
30	Oleanolic acid	5.99	C_30_H_48_O_3_	455.1524	455.0423	1.96	+	+	[36]

**Table 3:** Chemical characterization of Abees and A 195 polysaccharides hydrolysate.

No.	RT	Identification	Abees	A 195
1	7.913	Glyoxylic acid	-	1.15
2	16.877	D-Arabinose	3.586	2.83
3	17.398	D-Fructofuranose	4.36	-
4	17.596	1,2,3-Propanetricarboxylic acid	-	14.75
5	17.782	L-Rhamnose	4.88	1.81
6	18.424	L-Gluconic acid	-	3.62
7	18.489	D-Allopyranose	1.8	-
8	18.943	Fructose oxime	6.66	-
9	18.993	D-Psicose	5.53	0.58
10	19.287	D-Talopyranose	4.04	2.67
11	19.430	Glucose	4.96	13.88
12	19.723	Galactose	7.6	9.36
13	19.785	Galacturonic acid	-	4.02
14	20.052	Glucaric acid	17.82	17.98
15	20.450	Myo-Inositol	1.62	3.99
16	25.517	2-Deoxy-galactopyranose	-	1.30
17	25.975	D-Xylopyranose	2.42	-
18	26.392	D-Glucuronic acid	-	2.17
19	27.225	3-alpha-Mannobiose	2.33	1.66

## Data Availability

The data presented in this study are available in article.

## Supplementary Materials

The following supporting information can be downloaded at: https://www.mdpi.com/article/10.3390/metabo14040222/s1, Table S1: MS/MS fragmentation pattern of some tentatively identified compounds using Mass Hunter software program.
