# Supplementary material for: A Comparative Analysis of Polysaccharides and Ethanolic Extracts from Two Egyptian Sweet Potato Cultivars, Abees and A 195: Chemical Characterization and Immunostimulant Activities
Source: Metabolites. 2024 Apr 14;14(4):222. doi: 10.3390/metabo14040222 (PMC11051996; doi:10.3390/metabo14040222)
Supplement: Supplementary file 1 [file metabolites-14-00222-s001.zip › metabolites-2881778-supplementary.pdf]

Table S1: MS/MS fragmentation pattern of some tentatively identified compounds using Mass Hunter software program.

Compound name	Fragmentation spectrum
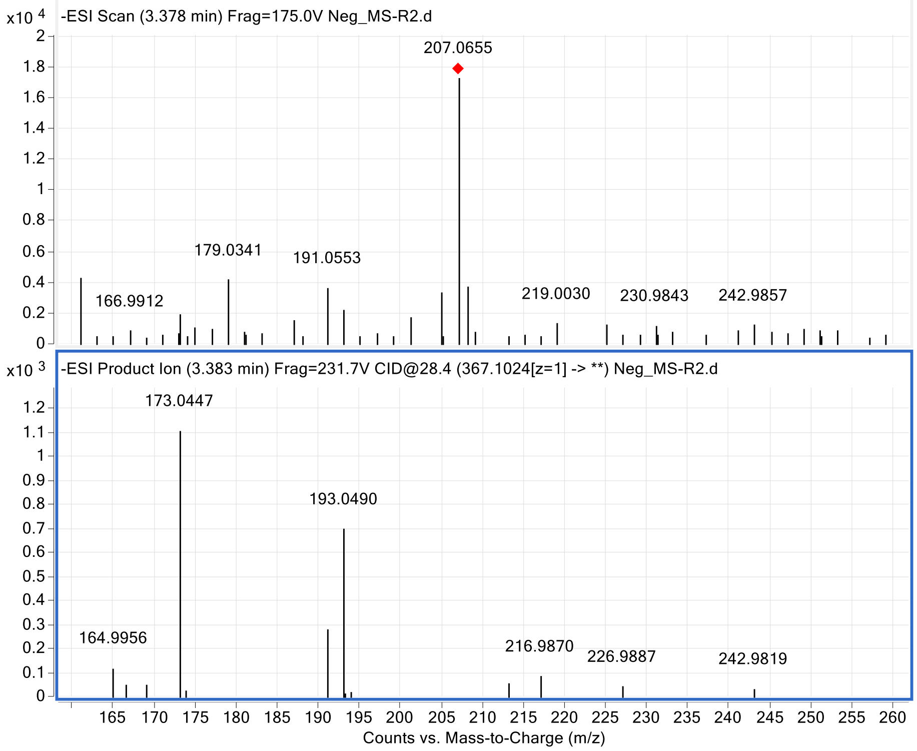	Feruloyl quinic acid
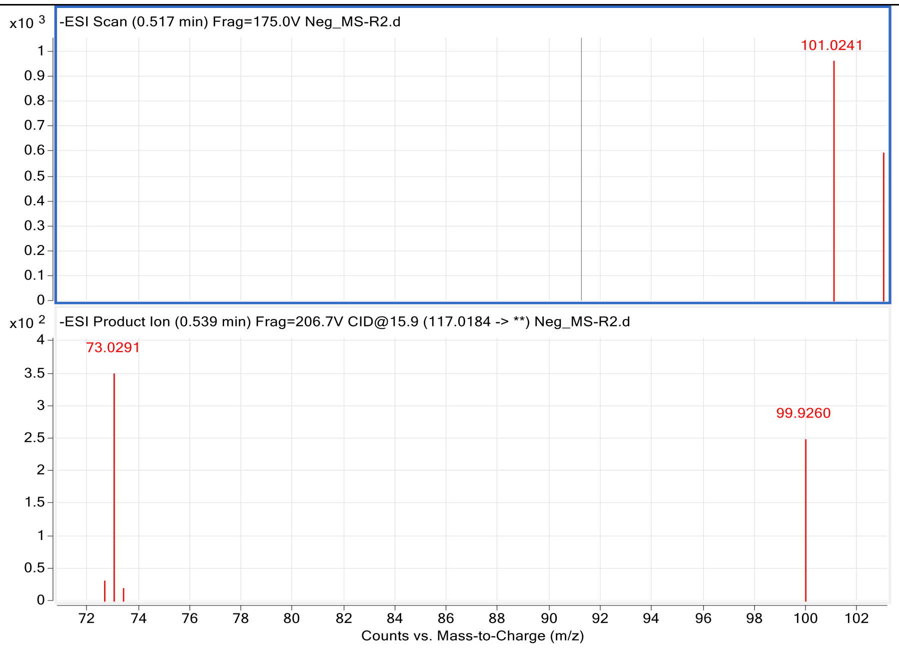	Succinic acid

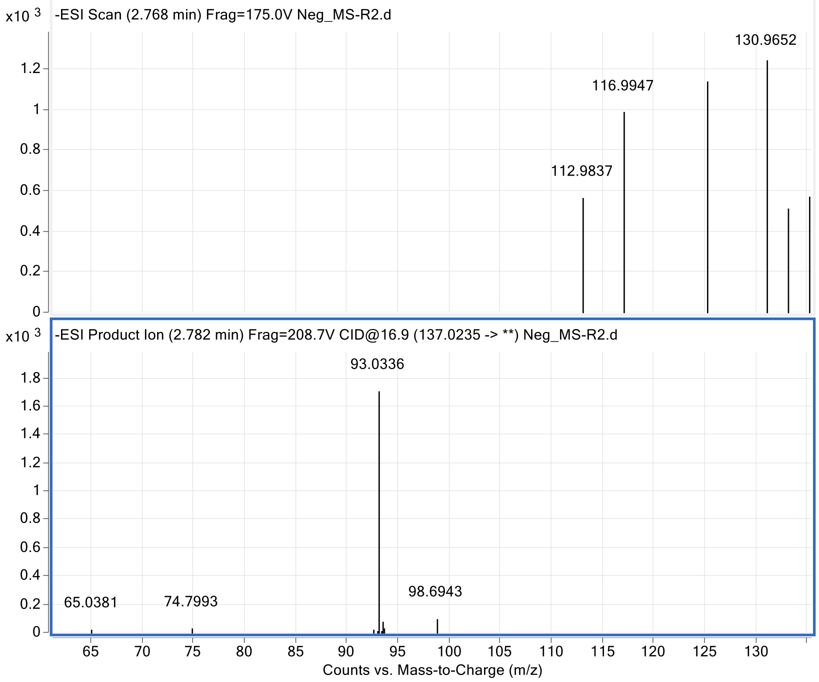	4-Hydroxybenzoic acid
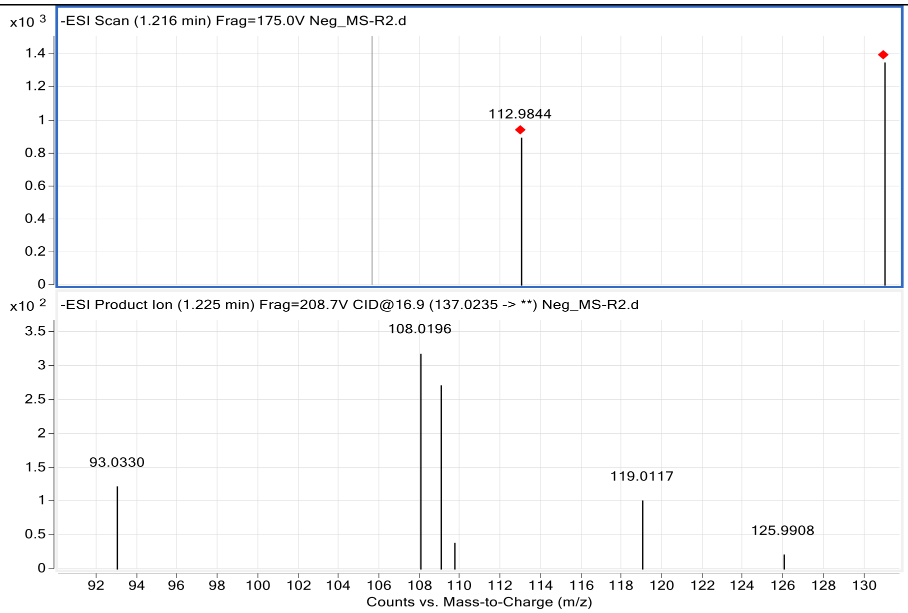	Protocatechualdehyde

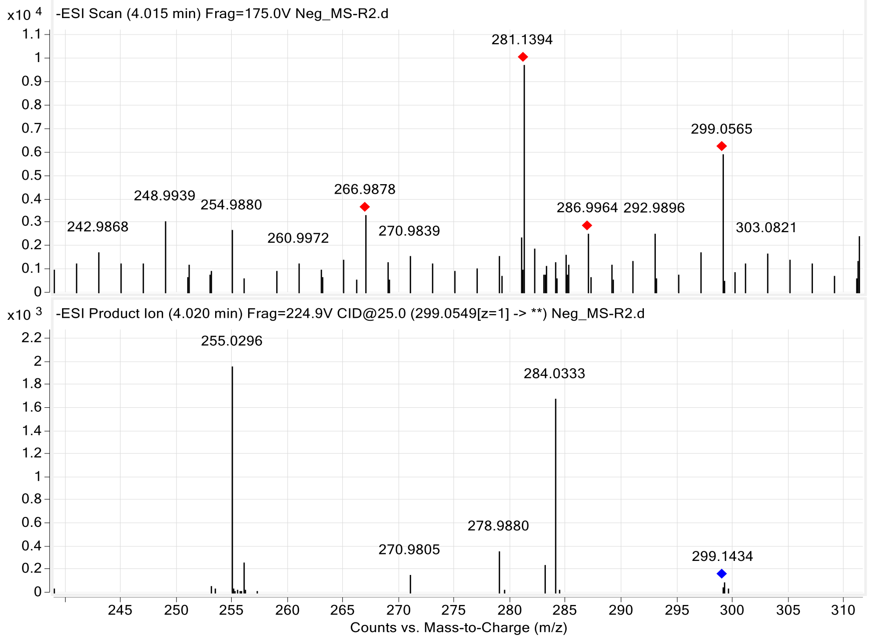x10 4<br>-ESI Scan (4.015 min) Frag=175.0V Neg_MS-R2.d<br>281.1394<br>299.0565<br>248.9939<br>254.9880<br>266.9878<br>286.9964<br>292.9896<br>242.9868<br>260.9972<br>270.9839<br>303.0821<br>x10 3<br>-ESI Product Ion (4.020 min) Frag=224.9V CID@25.0 (299.0549[z=1] -> **) Neg_MS-R2.d<br>255.0296<br>284.0333<br>278.9880<br>270.9805<br>299.1434<br>Counts vs. Mass-to-Charge (m/z)	Diosmetin
